# CRISPR-Cas9 Knock-In of T513M and G41S Mutations in the Murine β–Galactosyl-Ceramidase Gene Re-capitulates Early-Onset and Adult-Onset Forms of Krabbe Disease

**DOI:** 10.3389/fnmol.2022.896314

**Published:** 2022-05-10

**Authors:** Rima Rebiai, Emily Rue, Steve Zaldua, Duc Nguyen, Giuseppe Scesa, Martin Jastrzebski, Robert Foster, Bin Wang, Xuntian Jiang, Leon Tai, Scott T. Brady, Richard van Breemen, Maria I. Givogri, Mark S. Sands, Ernesto R. Bongarzone

**Affiliations:** ^1^Department of Anatomy and Cell Biology, College of Medicine, University of Illinois at Chicago, Chicago, IL, United States; ^2^Department of Pharmaceutical Science, College of Pharmacy, Oregon State University, Corvallis, OR, United States; ^3^Department of Medicine, Washington University School of Medicine, St. Louis, MO, United States; ^4^Department of Genetics, Washington University School of Medicine, St. Louis, MO, United States

**Keywords:** galactosylceramidase, demyelination, synapses, lysosome, autophagy

## Abstract

Krabbe Disease (KD) is a lysosomal storage disorder characterized by the genetic deficiency of the lysosomal enzyme β-galactosyl-ceramidase (GALC). Deficit or a reduction in the activity of the GALC enzyme has been correlated with the progressive accumulation of the sphingolipid metabolite psychosine, which leads to local disruption in lipid raft architecture, diffuse demyelination, astrogliosis, and globoid cell formation. The *twitcher* mouse, the most used animal model, has a nonsense mutation, which limits the study of how different mutations impact the processing and activity of GALC enzyme. To partially address this, we generated two new transgenic mouse models carrying point mutations frequently found in infantile and adult forms of KD. Using CRISPR-Cas9 gene editing, point mutations T513M (infantile) and G41S (adult) were introduced in the murine GALC gene and stable founders were generated. We show that *GALC*^*T*513*M*/*T*513*M*^ mice are short lived, have the greatest decrease in GALC activity, have sharp increases of psychosine, and rapidly progress into a severe and lethal neurological phenotype. In contrast, *GALC*^*G*41*S*/G41*S*^ mice have normal lifespan, modest decreases of GALC, and minimal psychosine accumulation, but develop adult mild inflammatory demyelination and slight declines in coordination, motor skills, and memory. These two novel transgenic lines offer the possibility to study the mechanisms by which two distinct GALC mutations affect the trafficking of mutated GALC and modify phenotypic manifestations in early- vs adult-onset KD.

## Introduction

Krabbe disease (KD) is a neuropathic lysosomal storage disease caused by deficiency of the enzyme β-galactosyl-ceramidase (GALC) (Suzuki and Suzuki, 1970). Loss-of-function of GALC blocks the lysosomal hydrolysis of galactosylceramides (GalCer) and galactosylsphingosine (psychosine) (Suzuki and Suzuki, 1970; Igisu and Suzuki, 1984; Kobayashi et al., 1987; Greiner-Tollersrud and Berg, 2000/2013; Wenger, 2001). KD is primarily a childhood disease with most patients presenting neurological symptoms soon after birth. Krabbe infants can develop irritability, spasticity, seizures, muscle weakness, and a progressive loss of motor and cognitive functions (Escolar et al., 2016). Late-onset forms of the disease develop usually beyond 7–10 years of age, are less frequent, and characterized by vision problems, walking difficulties, and cognitive deterioration (Graziano and Cardile, 2015). The abnormal buildup of psychosine in the central and peripheral nervous systems is considered the main contributor to neuropathology in infantile severe forms of KD. Psychosine promotes demyelination (Taniike and Suzuki, 1994; Suzuki and Taniike, 1995; O’Sullivan and Dev, 2015), gliosis and inflammation (Giri et al., 2002), disruption of lipid rafts (White et al., 2009; Hawkins-Salsbury et al., 2013), neuropathy (Castelvetri et al., 2011, 2013; Smith et al., 2011; Cantuti-Castelvetri et al., 2012, 2015), and alterations in many signal transduction pathways (Giri et al., 2008; Castelvetri et al., 2013; Sural-Fehr et al., 2019). While the pathogenic role of psychosine in infantile KD is well documented, its contribution to neuropathology in adult forms of KD is poorly understood, primarily due to the lack of adult-onset KD animal models and mechanistic studies in patients with this form of the disease.

The most widely used KD animal model is the Twitcher (TWI) mouse, in which a naturally occurring mutation leads to degradation of GALC mRNA and absence of functional GALC protein (Duchen et al., 1980; Lee et al., 2006). The TWI mouse is a surrogate model of KD, with many hallmarks observed in human patients with infantile KD. These include toxic accumulation of psychosine, ataxic gait deficits, muscular weakness, hind limb paralysis, and are short life span. Other animal models, including the globoid cell leukodystrophy (GLD) dog, the non-human primate, and various murine lines (Bradbury et al., 2020), also largely model severe forms of KD. There are no described animal models of adult-onset KD.

To contribute to the study of neuropathic mechanisms of disease in KD, particularly those at play in adult-onset forms, we used CRISPR-Cas9 gene editing to introduce early infantile and adult-onset KD point mutations in the GALC gene. Over 140 mutations and polymorphisms in the GALC gene have been described (Wenger et al., 1997; Tappino et al., 2010; Graziano and Cardile, 2015) with the 30 kb deletion of exons 10–17 being frequently found in severe infantile forms. Previous *in vitro* studies showed how several point mutations in the GALC gene, found in homozygous as well as in compound heterozygous patients, can affect not only GALC activity but also its trafficking to the lysosomes (Lee et al., 2010; Shin et al., 2016; Irahara-Miyana et al., 2018). Based on this, we have selected point mutations T513M, present in many infantile patients (Wenger et al., 1997) and G41S, present in many adult patients (Lissens et al., 2007), to generate knock in mouse models. This report presents the characterization of *GALC*^*T*513*M/T*513*M*^ and *GALC*^*G*41*S*/G41*S*^ transgenic mice, which recapitulate early-onset (*GALC*^*T*513*M/T*513*M*^) and late-onset (*GALC*^*G*41*S*/G41*S*^) KD forms.

## Materials and Methods

### CRISPR Mouse Construction: Zygote Injection of CRISPR Guide RNA, Cas9 RNA, and Homology Directed Repair Single Strand DNA Oligo Template

All procedures involving animals were conducted in accordance with protocols approved by the Animal Care and Use Committee at the University of Illinois at Chicago (Protocol Number 15-101). CRISPR reagents were produced at the UIC Genome Engineering Core Facility. We use the legacy nomenclature for identifying mutations.

#### sgRNA Plasmid Construction

Two Cas9-sgRNA expression plasmids were constructed by ligating oligonucleotide duplex with the forward sequence 5′-GTCTAGCACGTAGGCGCCAC-3′ and 5′-GAACTTGGCGCAGCGTGAAG-3′ into *Bsa*I cut addgene plasmid DR274, which was a gift from Keith Joung (Addgene plasmid #42250). The sgRNA target sequences were designed to target the GALC gene near codons G41 and T528, respectively using the MIT CRISPR design algorithm.

#### Homology Directed Repair Donor Construction

A single strand oligo consisting of 70 bp homology arms flanking the two desired mutations G41S and T528M was synthetized (IDT). The Sequences were as follows:

##### ssODN Donor Mutation G41S: GGC->AGC (Bold)

5′-TGACCGCCGCCGCGGGCTCGGCGAGCCGTGTTGCGG TGCCCTTATTGTTGTGTGCGCTGCTAGTGCCTGGT**AGC**G CCTACGTGCTAGACGACTCCGACGGGCTGGGCCGGGAG TTCGATGGCATCGGCGCAGTCAGCGGCGGCGGG-3′

##### ssODN Donor Mutation T513M: ACG->ATG (Bold)

5′-GCTCCGAATTTTGCTGATCAGACTGGCGTGTTTGAGT ACTACATGAATAATGAAGACCGTGAGCAACGCTTC**ATG**C TGCGCCAAGTTCTCAACCAACGACCTATTACCTGGGCTG CAGACGCTTCCAGCACAATCAGTGTT-3′

#### Guide RNA Production and Validation

*In vitro* transcription of RNA was performed using the MEGAshortscript™ T7 Transcription Kit. RNA function was validated with an *in vitro* cas9 Digestion assay in which the target region DNA was amplified from black 6 mouse genomic DNA and allowed to incubate with cas9 nuclease protein from NEB (M0386T) and the transcribed Guide RNA. Mutations were confirmed by next generation sequencing before zygote injections.

#### Mouse Zygote Injections

Injection of CRISPR reagents was performed at University of Chicago Transgenic Core following the protocol as described (Wang et al., 2013). Pups were genotyped at UIC using a next generation amplicon sequencing technique which is able to quantify the efficiency of the CRISPR edits in each individual mouse (Cong et al., 2013; Hsu et al., 2013).

### Generation of Transgenic Mice

**Key:** Gray is exon sequence**. Bold** is sequence where sgRNA binds. Double strand break occurs at ^^^^^^.

#### G41S: GGC to Be Changed to AGC

5′TAATTACACGCAGAGACCGGTCCCGCCTCTTTGACACA GAAGTGACAAGGCAAAGCTCGCTCAACCAGCCCCCCAC CCCGCCCCCCCAGCTCAACACAACAGCGGCTGCGCGGA CAGCCCGCAGCCCTCACTTAAGATGGCGAAAGCTTCCT CAGCCGCCCGCTCTTCTTCCTCAGGGAGGCGATCGGGC CCGCCTCCCCGGGCGCCACAGTCGCGTGACCCGCACAA TGGCTAACAGCCAACCTAAGGCTTCCCAGCAACGCCAA GCAAAAGTCATGACCGCCGCCGCGGGCTCGGCGAGCC GTGTTGCGGTGCCCTTATTGTTGTGTGCGCTGCTAGTG C**CCGGTG**^^^^^^**GCGCCTACGTGCTAGAC**GACTCCGACG GGCTGGGCCGGGAGTTCGATGGCATCGGCGCAGTCAG CGGCGGCGGGGTGAGCGCGGGCTGGCGGGAGGCAGGA GTGCGGTGCGGCGCAGGGACCGCAGGGACCGCAGGGA CCGCCGGGGCCGCCCCCTCCCACAAGCCCCCGCGGCGT TGCCGGGCGAGCGCACGCCGCTTCCCCGCGCGCCGGG GTGAGATGAACCCGGGCTGGCGGTTAGATTGCAATGGG GAGCCAGGCGTCTCGAGGGGACAGGCGTCTGCTGCAG TCAAGTGGCCCGGGCTCGAGGGAGCCCTCGTTCTGGAT CGCCGGC-3′

#### G41S Donor Template for HDR

5′-TGACCGCCGCCGCGGGCTCGGCGAGCCGTGTTGCGG TGCCCTTATTGTTGTGTGCGCTGCTAGTGC**CtG**GTaGCGC CTACGTGCTAGACGACTCCGACGGGCTGGGCCGGGAGTT CGATGGCATCGGCGCAGTCAGCGGCGGCGGG-3′

*Kas*I site was destroyed when the mutation was made, which can be used to screen mice. PAM site was mutated to avoid cutting donor template by guide (Silent Mutation *P* = CCC -> *P* = CCt).

#### T513M: ACG Changed to ATG

5′-TACAGGCATCTGGTCAATTATTTGGATATATTTCCAAA TACATCACACACACACACACACACACACCCTAAACTTTG TTTCTAAGTATTATGTTTTATAAATTAGTCAAGTATTTCA CAGTGTATCACCGATATCAGAAAGGAGAGATCTGGTAAG GGCTTGCTAATCAGCCGCTGAGATACTAAGTGGAGGACT TACTTTTTGTGTTCTGAACAGAGTACCCACTTTTTAGTG AAGCTCCGAATTTTGCTGATCAGACTGGCGTGTTTGAGT ACTACATGAATAATGAAGACCGTGAGCA**CCGCTT**^^^^^^**C ACGCTGCGCCAAGTTC**TCAACCAACGACCTATTACCTG GGCTGCAGACGCTTCCAGCACAATCAGTGTTATAGGCGA TCACCACTGGTGTGTGAGAGATGCCCTCAGTGTATACGC TTGCGATTTGGGGTACATTCTGAGTGAAGGGATTCCAA TAGGCCGTCTCCTGTCAAGAACCTAATAGGTGGTGGGTC AGAATTATGTGACCTCAAGTGATCAAATTAGCTTCCATG TCCTCCCTTTAGATTATGCCAGTTTGAAGATAATTGCGG TCAGAGAAATCCAACTTTAATGGGAAAATTGAGG-3′

#### T513M Donor Template

5′GCTCCGAATTTTGCTGATCAGACTGGCGTGTTTGAGT ACTACATGAATAATGAAGACCGTGAG**CAaCG**CTTCAtGCT GCGCCAAGTTCTCAACCAACGACCTATTACCTGGGCTGC AGACGCTTCCAGCACAATCAGTGTT-3′

*Sdu*I restriction enzyme site was created when the mutation was made, which can be used to screen mice. PAM site was mutated to avoid cutting donor template by guide (Silent mutation H = CAC –> H = CAa).

### Western Blot Analyses

Total brain tissues were homogenized and prepared in RIPA buffer supplemented with phosphatase and protease inhibitors. Protein concentration analysis was determined with a BCA protein assay kit (Thermo Fisher Scientific, Waltham, MA, United States) according to manufacturer’s instructions. Samples were prepared with 6x Laemmli buffer and lysis buffer. Twenty five micrograms of protein were loaded per lane and separated by 12% SDS-PAGE under denaturing conditions with a mini-Protean II gel electrophoresis apparatus and included a Precision Plus Standard Protein Dual color (Biorad) to enable identification of band size. Separated proteins were then transferred to a PVDF membrane. Non-specific binding sites on the membrane were blocked with 5% skim milk in TBS-Tween 20 for 1 h at room temperature. Primary antibodies against GALC (in house produced monoclonal mouse IgG, 1:100 dilution), myelin proteolipids (PLP, a kind gift from Robert Skoff, Wayne University, rabbit IgG, 1:2,000 dilution), myelin basic proteins (MBP, a kind gift from Anthony Campagnoni, UCLA; rabbit IgG, 1:500 dilutiom), Actin (Sigma-Aldrich, rabbit, 1:2,000), LAMP1, LAMP2 (Iowa Hybridoma Cell Bank, mouse IgG, 1:500) and LC3 (Cell Signal, rabbit IgG, 1:500) were prepared in 5% milk in TBS/Tween 20 and incubated at 4°C overnight. Membranes were washed in TBST (0.05%) 3× for 5 mins and incubated for 1 h at room temperature with secondary antibody. Secondary antibodies were horseradish peroxidase-conjugated (HRP), diluted in 5% milk TBST, and then developed using the Odyssey CLx apparatus (Li-Cor). Protein band densities were quantified using ImageJ and normalized by the housekeeping protein GAPDH.

### Immunohistochemistry

Mice were perfused with PBS under 5% isoflurane anesthesia followed by 4% paraformaldehyde (PFA) before tissue was removed and processed for cryosectioning. Cryosections of 30 μm thickness were cut for immunohistochemistry (IHC). The sections were blocked free-floating with blocking buffer (0.3 M glycine, 1% BSA, 5% normal donkey serum, 5% normal goat serum, 0.30% Triton X-100, TBS) for 1 h at room temperature, followed by 24–72 h incubation at 4°C with primary antibodies in blocking solution. After washing with TBS, tissue was incubated with secondary antibodies at room temperature for 1 h in blocking buffer and washed again in TBS. Tissue was mounted with Prolong Gold antifade reagent (cat# P36931 Life technologies, Eugene, OR, United States) and visualized using confocal microscopy (Leica TCS SPE, Wetzlar, Germany). Primary antibodies used included: GALC (in house produced mouse IgG monoclonal, 1:100 dilution), PLP (a kind gift from Robert Skoff, Wayne State Univ, rabbit IgG 1:2,000 dilution), GFAP (Millipore, Mouse, 1:1,000), IBA-1 (Millipore, rabbit IgG 1:300), LAMP1 and LAMP2 (Iowa Hybridoma Cell Bank, mouse IgG, 1:500). Secondary antibodies used included: AlexaFluor 488 anti-Mouse (cat#A-11029, Thermo Fisher Scientific, Waltham, MA, 1:500 dilution) and Dylight 549 anti-rat (cat# 112-506-068 (Jackson ImmunoResearch, 1:500 dilution). Counterstaining for cell nuclei was performed with DAPI (4′,6-diamidino-2-phenylindole, cat#D1306, Thermo Fisher Scientific, Waltham, MA, 1: 3,000 dilutions in TBS). Immunofluorescent complexes were visualized using a Leica TCS SPE confocal laser with an upright DM5500Q Microscope (Leica Biosystems Inc., Buffalo Grove, IL, United States). Counting of GALC/Lamp double positive puncta were done by confocal microscopy with an immersion oil 63x objective on four random fields (*n* = 3 mice/condition) and Image J-Graph Pad *post hoc* analyses.

### β−Galactosyl−Ceramidase Activity and Quantification of Glucosylsphingosine and Psychosine

Our methods have been described in detail in Marshall et al. (2018). To summarize, a Vibra-cell ultrasonic liquid processor model#VCX 130 (Sonics and Materials Inc., Newton, CT, United States) was used to homogenize fresh frozen tissue in water. Fluorescent GALC substrate (6HMU-beta-D-galactoside; Moscerdam Substrates) was incubated with tissue lysates (20 μg) for 17 h at 37°C before the reaction was stopped. Fluorescence was measured to assess enzyme activity using a Beckmann Coulter DTX 880 multimode detector (Beckman Coulter, Brea, CA, United States) through excitation/emission wavelengths of 385 and 450 nm, respectively. Methanol-acetic acid solution (0.5% Acetic Acid in methanol) was used to extract psychosine from tissue homogenates (brain and spinal cord lysates) (200 μg). Psychosine content was then determined by liquid chromatography-tandem mass spectrometry (LC-MS/MS). To differentiate glucosylsphingosine vs psychosine, the following protocol was used: mouse brains were homogenized in 2% CHAPS solution (4 mL/g brain). Protein precipitation was used to extract psychosine from 50 μL of homogenate in the presence of d5-psychosine and d5-glucosylsphingosine as internal standard for psychosine and glucosylsphingosine, respectively. Quality control (QC) samples were prepared by pooling 10% of extracts from study samples. The QC samples were used to monitor the instrument performance and injected every five study samples. Analysis of psychosine and glucosylsphingosine was performed with a Shimadzu 20AD HPLC system coupled to a 4000QTrap mass spectrometer equipped with an electrospray ion source and operated in positive multiple reaction monitoring (MRM) mode (Sidhu et al., 2018). Data processing was conducted with Analyst 1.6.3 (Applied Biosystems). The relative quantification of psychosine and the data were reported as the peak area ratios of the psychosine to its internal standard.

### Monoclonal β−Galactosyl−Ceramidase Antibody Generation

Monoclonal mouse anti-GALC antibodies were generated with the goal to recognize full-length human and mouse GALC proteins in tissues and protein extracts. Monoclonals were prepared following procedures described previously (Pfister et al., 1989). In short, mice were immunized subcutaneously two times with 25 μg of a keyhole limpet hemocyanin-conjugated synthetic peptide encompassing aminoacids 469–498 of human GALC (which shares >90% homology with the mouse protein sequence), mixed with Gerbu adjuvant. Before 3 days of fusion, the mice received an intravenous injection with 10 μg of antigen together with adjuvant. Spleen and lymph node cells were fused with SP2 myeloma cells to generate hybridomas. Positive clones were selected and cloned by repeated screening against the GALC protein using enzyme-linked immunosorbent assay (ELISA). The performance and specificity of several antibody clones were validated using western blot and immunofluorescence microscopy against transfected cells.

### Disease Severity Score

The clinical conditions of the mice were assessed and quantified using an expanded disease severity score (DSS) based on our previous DSS system (Marshall et al., 2018). The new scoring system takes into consideration the following general categories: physical characteristics (weight loss, tremor, and kyphosis), locomotion, wire hanging, and cerebellar function (hindlimb clasping, ledge balance). Each individual test is scored as follows: weight loss: compared to average of previous 2 weeks score of 0: 0.5 loss or less (including weight gain); score of 0.5: 0.5–1 g loss; score 1: 1–1.5 g loss; score 1.5: 1.5–2 g loss; score 2: >2 g loss. Tremor score 0: no tremor; score; score 1: mild tremor; score 2: severe tremor. Kyphosis score 0: no kyphosis in any condition; score 1: kyphosis when hunched over; score 2: kyphosis when lying flat. Locomotion score 0: normal; score 0.5: awkward gait; score 1: waddling; score 1.5: one leg paralyzed; score 2: both legs paralyzed. Wire hanging score 0: >45 s; score 0.5: 31–45 s; score 1: 16–30 s; score 1.5: 5–15 s; score 2: 0–5 s. Hindlimb clasping score 0: none; score 0.5: one limb partial (under 5 s out of 10); score 1: two limbs partial or 1 limb full (over 5 s out of 10); score 2: both limbs full. Ledge balance score 0: no problems; score 0.5: minor problem (mouse is reaching down and slips); score 1: minor fall (mouse is starting to move down and falls); score 2: fall from ledge with no attempt to get down.

### Motor and Behavioral Tests

Latencies to fall in the rotarod below normal values were considered pathognomonic and measured as described (Marshall et al., 2018). Motor activity in the open field test was evaluated as described (Kan et al., 2016; Nelvagal et al., 2021). Mice were tested for anxiety using the light/dark box (Malmberg-Aiello et al., 2002; Bourin and Hascoet, 2003). Spatial memory was evaluated using the Barnes and Morris water maze tests as described (Patil et al., 2009).

### Neural Precursor Cultures and Starvation Model

Neural precursors were isolated from the subventricular zone from P4 to 6 mice and stable self-proliferating cultures were established as described (Felling et al., 2006; Givogri et al., 2008). Cells were differentiated on poly-lysine coated coverslips in defined medium containing 1% FBS for 7 days. Half of the coverslips were replenished with complete medium containing FBS and the other half with FBS-depleted medium. Starvation (Mejlvang et al., 2018) was then maintained for 4 h before cells were washed and fixed in 1.5% PFA/PBS for 30 min. Cells were processed for double immunohistochemistry using anti-LAMP-2 (see above) and anti-LC3 (see above) antibodies. After mounting and nuclear staining using DAPI, confocal images were taken using a 63x oil objective. Double LAMP-2+/LC3+ (i.e., colocalization and yellow fluorescence) and single LAMP-2+ puncta were counted in 15–20 cells per condition.

### Statistical Analysis

Prism 8 software (GraphPad Software Inc., La Jolla, CA, United States) was used to prepare graphs and statistics. One-way ANOVA with Gaussian distribution was performed to analyze data with more than two means, with two-sided *p*-values < 0.05 considered significant. *T*-tests were also performed for the comparisons between two means, with two-sided *p* < 0.05 considered significant. Graphs represent the mean of independent measurements (with sample sizes ranging *n* = 6–12) shown with errors bars representing standard error of the mean.

## Results

### *Biochemically, GALC*^*T*513*M*/*T*513*M*^ and *GALC*^*G*41*S*/G41*S*^ Mice Largely Recapitulate Early-Onset and Late-Onset Krabbe Disease, Respectively

**FIGURE 1:**
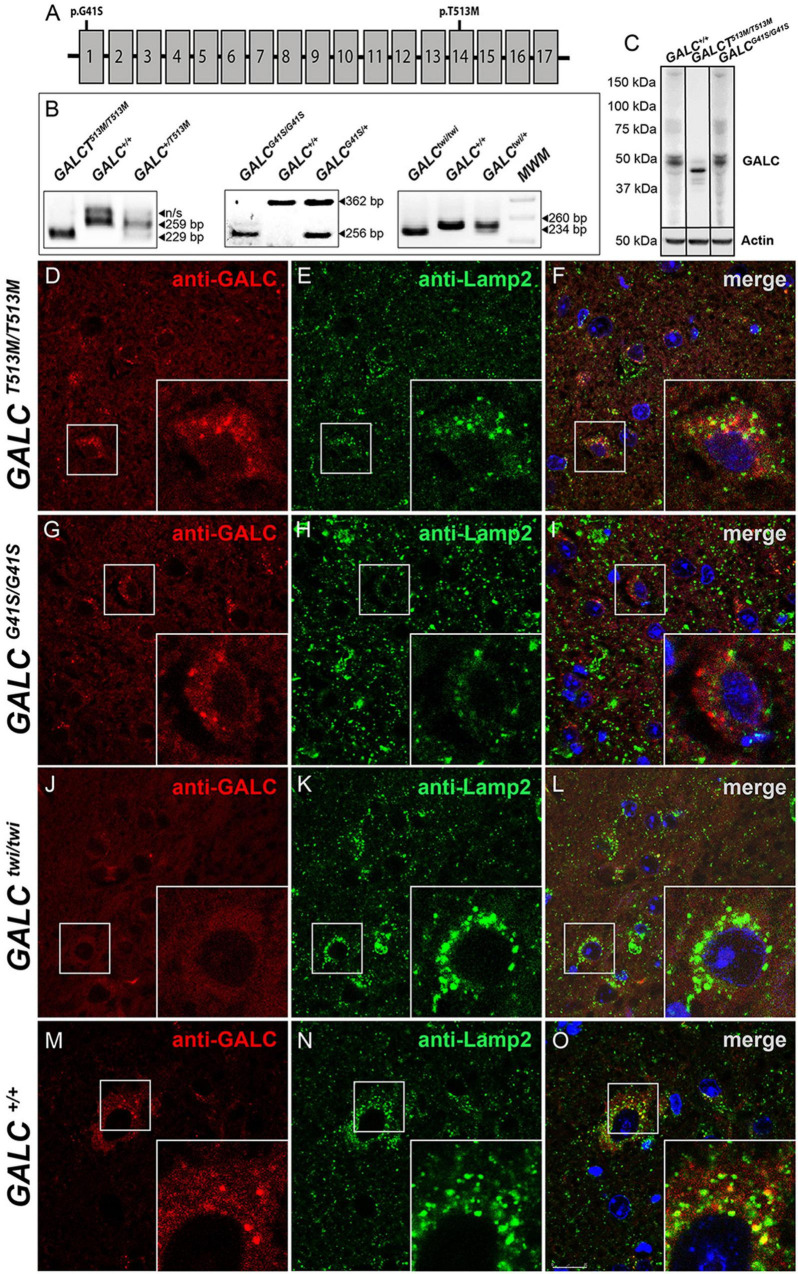
Lysosomal localization of mutated GALC protein is reduced in *GALC*^*T*513*M/T*513*M*^ and GALC^*G41S/G41S*^ mice. **(A)** Localization of the G41S and T513M mutations within the *GALC* gene. **(B)** PCR-based identification of mutations in *GALC*^*T*513*M/T*513*M*^ and GALC^*G41S/G41S*^. Mutation in *GALC*^*twi/twi*^ and GALC^+/+^ were used as references. **(C)** Western blot analysis of GALC protein lysates prepared from P40 brains. Actin was used for loading control. **(D–O)** Immunohistochemistry on cryosections of P25 brain tissue showing colocalization of GALC protein (red) with LAMP-2 (green) in lysosomes of *GALC*^*T*513*M/T*513*M*^, GALC^*G41S/G41S*^, GALC^*twi/twi*^, and GALC^+/+^ mice. DAPI, blue. Scale bar = 50 μm.

**FIGURE 2:**
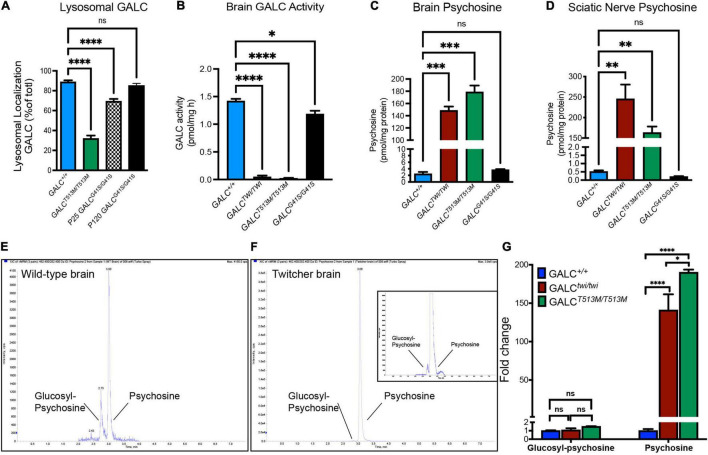
Analysis of mutated GALC activity and psychosine production in *GALC*^*T*513*M/T*513*M*^ and GALC^*G41S/G41S*^ mice. **(A)** Co-localization of GALC in lysosomes was measured by confocal analysis of brain cortices of each genotype. Data represent the fraction (as%) of total GALC+ puncta that was co-stained with Lamp2+. Counts were done on random areas of the brain cortex. **(B)** GALC activity was analyzed fluorometrically in protein extracts from P40 brains. **(C,D)** Psychosine levels in P40 brains **(C)** and sciatic nerve **(D)** were measured by LC-MS-MS. **(E–G)** Chromatograms of glucosylsphingosine and psychosine in WT **(E)** and twitcher mouse brains **(F)** were detected by mass spectrometry. The glucosylsphingosine and psychosine were measured as the fold changes in the brain of Twi and GALC^*T*513*M/T*513*M*^ mutants versus WT **(G)**. Data represent the mean ± SEM of 4–6 mice/genotype, analyzed by ANOVA/Tukey’s *post hoc* test *p < 0.05, ^**^p < 0.01, ^***^p < 0.001, ^****^p < 0.001.

The presence of the *GALC*^*T*513*M*/*T*513*M*^ and *GALC*^*G*41*S*/G41*S*^ mutations (Figure 1A) was confirmed by PCR analysis from total genomic DNA (Figure 1B). Western blotting analysis of protein extracts prepared from P25 brains indicated that mutated GALC proteins are clearly expressed in both *GALC*^*T*513*M/T*513^ and *GALC*^*G*41*S*/G41*S*^ mutants, although the GALC^*T*513*M*^ protein appears to have a lower molecular weight (Figure 1C), which is likely indicating abnormal glycosylation of the mutated protein. Studies have previously reported that many point mutations in the GALC protein alter the transport to the lysosomes (Lee et al., 2010; Shin et al., 2016; Irahara-Miyana et al., 2018). To examine whether this occurs in *GALC*^*T*513*M/T*513^ and *GALC*^*G*41*S*/G41*S*^ mutants, immunofluorescence confocal microscopy was used to determine if the mutated GALC protein colocalized with the lysosomal marker LAMP-2. Brain sections of both mutants were analyzed at postnatal day 25 (P25), using aged-matched Twi and WT as references of absence and normal presence of GALC protein, respectively (Figures 1D–O). Remarkably, the analysis showed reduced co-staining for GALC^*T*513*M*^ and GALC^*G*41*S*^ mutant proteins with Lamp2 (Figures 1F,I), suggesting partial mislocalization with lysosomes. As expected, TWI mutants had no immunodetectable GALC protein (Figure 1L) in comparison with wild-type controls (Figure 1O). Quantification of double positive GALC/LAMP-2 puncta showed ∼70% less *GALC*^*T*513*M*/*T*513*M*^ protein located in lysosomes (Figure 2A). Interestingly, association of *GALC*^*G*41*S*/G41*S*^ protein with LAMP-2+ puncta was reduced at early times points (P25) but not in older mice (P120, Figure 2A).

Next, we assessed how *GALC*^*T*513*M/T*513^ and *GALC*^*G*41*S*/G41*S*^ mutations impacted enzyme activity. As expected for an infantile mutation, mice with the *GALC*^*T*513*M/T*513^ mutation have undetectable enzyme activity in the brain, similar to levels in the TWI mouse (Figure 2B). In contrast, analysis of GALC activity in the brain of *GALC*^*G*41*S*/G41*S*^ mice showed a small reduction in enzyme activity (Figure 2B).

The loss of function of GALC enzyme in KD results in the progressive accumulation of psychosine (Igisu and Suzuki, 1984; White et al., 2009; Spassieva and Bieberich, 2016). To examine how these mutations impacted the accumulation of psychosine, lipid extracts from P40 *GALC*^*T*513*M/T*513^, *GALC*^*G*41*S*/G41*S*^, and TWI brain (Figure 2C) and sciatic nerves (Figure 2D) were analyzed by LC-MS-MS. Consistent with the lack of GALC activity, both *GALC*^*T*513*M*/T513*M*^ and TWI tissues have the highest levels of psychosine (Figures 2C,D). In contrast, *GALC*^*G*41*S*/G41*S*^ mice showed psychosine levels similar to wild type in both central and peripheral nerve tissue. To confirm the accumulation of psychosine in *GALC*^*T*513*M/T*513^ mice, we measured the relative levels of glucosylsphingosine and psychosine, using a LC-MS/MS protocol to completely separate the isomeric glucosylsphingosine and psychosine (glucosylsphingosine and psychosine have the same *m/z* 462) (Figure 2E). As expected, both TWI and *GALC*^*T*513*M/T*513^ mutants accumulate high levels of psychosine, while glucosylsphingosine is only found at trace levels (Figures 2E,F). Figure 2G shows the relative abundance of psychosine and glucosylsphingosine in TWI and *GALC*^*T*513*M/T*513^ brains versus WT brains, confirming that only psychosine is accumulated in both mutants. Interestingly, psychosine in TWI is higher than in *GALC*^*T*513*M/T*513^ brains.

### *GALC*^*T*513*M/T*513*M*^ and *GALC*^*G*41*S*/*G*41*S*^ Mice Develop Clinical and Motor Phenotypes Consistent With Early-Onset and Late-Onset Krabbe Disease, Respectively

**FIGURE 3:**
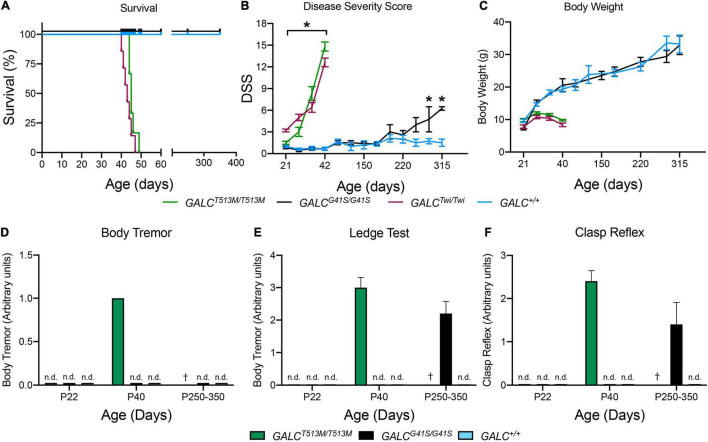
Clinical analysis of *GALC*^*T*513*M/T*513*M*^ and GALC^*G41S/G41S*^ mice. Survival **(A)**, disease severity score (DSS) **(B)**, body weight **(C)**, onset of body tremor **(D)**, onset of errors on the ledge test **(E)**, and onset of affected clasp reflex **(F)** were measured for each genotype. n.d., non-detected; †, dead. *n* = 6–8 mice/genotype/time point. *p < 0.05.

*GALC*^*T*513*M/T*513^ mice have a median survival of 46 vs 42 days in Twi mice (Figure 3A). The maximal survival of *GALC*^*T*513*M/T*513^ was 49 ± 3 days. In contrast, *GALC*^*G*41*S*/G41*S*^ mice have no significant decrease in their survival, with all mutants reaching adult age similar to WT mice (Figure 3A). Expectedly, *GALC*^*T*513*M/T*513^ mice and *GALC*^*G*41*S*/G41*S*^ mice show remarkably different clinical signs of neurological disease. Using our established DSS system (Marshall et al., 2018), *GALC*^*T*513*M/T*513^ mice have an onset with significant increases in DSS at postnatal day 24 (P24 ± 2 days). This is slightly delayed in comparison with Twi mice, which show clinical onset at P22 ± 2 days (Figure 3B). *GALC*^*T*513*M/T*513^ mice follow a typical rapid and progressive increase in DSS, similar to TWI mice (Figure 3B). In contrast, *GALC*^*G*41*S*/G41*S*^ mice show DSS values comparable to WT for most of their life until approximately 250 days, when minor but significant motor deficits were measured (Figure 3B). In terms of body weight, *GALC*^*T*513*M/T*513^ mice and *GALC*^*G*41*S*/G41*S*^ mice showed expected decreases and normal gains, respectively (Figure 3C).

As expected for a severe demyelinating phenotype, *GALC*^*T*513*M/T*513^ mice showed increased tremor by P40 (Figure 3D). *GALC*^*G*41*S*/G41*S*^ mice did not have detectable tremor at any time point in our study (Figure 3D). Assessment in the ledge test showed an early deficit in *GALC*^*T*513*M/T*513^ mice at ∼P40 (Figure 3E). Interestingly, *GALC*^*G*41*S*/G41*S*^ mice did not have measurable deficits in this test until they reached adulthood (P250–350) (Figure 3E). Similarly, a positive clasping test was detected in ∼P40 *GALC*^*T*513*M/T*513^ mice and only in aged (>P250) *GALC*^*G*41*S*/G41*S*^ mice (Figure 3F).

**FIGURE 4:**
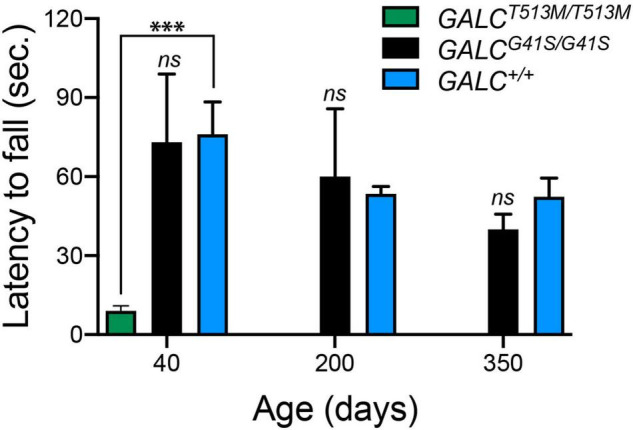
*GALC*^*T*513*M/T*513*M*^ but not GALC^*G41S/G41S*^ mice exhibit rotarod deficits. Rotarod performance was significantly lower in *GALC^T513M/T513M^* mutants. Results are expressed as latency to fall in seconds (sec). Mice were tested at P40, P200, and P350. Data represent the mean + SEM of *n* = 6/group, analyzed by ANOVA/Tukey’s *post hoc* test ^***^p < 0.01.

To further evaluate motor performance and balance, *GALC*^*T*513*M/T*513^ and *GALC*^*G*41*S*/G41*S*^ mice were trained using an accelerating rotarod test. As expected for animals with a severe neurological phenotype, P40 *GALC*^*T*513*M/T*513^ mice showed a significantly impaired capacity to remain in the rotarod (Figure 4). In contrast, *GALC*^*G*41*S*/G41*S*^ mice had no significant deficits in their latency to fall from the rod either when young (P40) or older (P200 and P350) (Figure 4).

**FIGURE 5:**
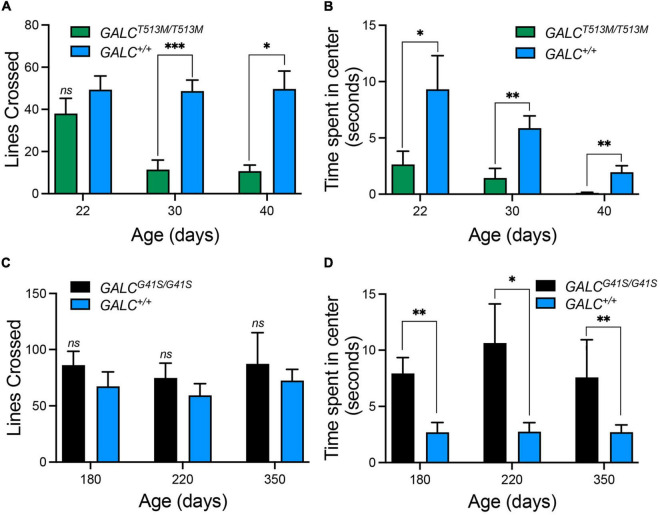
Distinct exploratory behavior and anxiety-type responses exhibited by *GALC*^*T*513*M/T*513*M*^ but not GALC^*G41S/G41S*^ mice in the open field test. **(A)** Progressive loss of motor exploratory activity (measured as number of lines crossed) was observed in *GALC^T513M/T513M^* mice, starting at 30 days. **(B)** Signs of increased anxiety type responses (measured as time spent in the center quadrant) in *GALC^T513M/T513M^* mice were detected immediately after weaning. **(C)** *GALC^G41S/G41S^* mice exhibited normal motor activity at all time points. **(D)** *GALC^G41S/G41S^* mice showed signs of reduced anxiety-type behavior. Data represent the mean + SEM of *n* = 6–8/group, analyzed by ANOVA/Tukey’s *post hoc* test *p < 0.05, ^**^p < 0.01, ^***^p < 0.005.

Next, we tested both mutants in the open field locomotor test. Line crosses and time spent in center were both calculated to examine motor behavior and anxiety. Figure 5A shows that *GALC*^*T*513*M/T*513^ mice display a progressive decline in mobility starting at P30. Exploratory and anxiety behaviors, measured as the time that the animal spent in the center quadrant of the open field, showed the expected reduced time when *GALC*^*T*513*M/T*513^ mice became less mobile at P30 and P40 (Figure 5B). However, younger P22 *GALC*^*T*513*M/T*513^ mice, which showed no obvious mobility limitations, also displayed a reduced time spent in the center quadrant. This suggests early signs of decreased exploratory behavior and likely, elevated anxiety in *GALC*^*T*513*M/T*513^ mice compared with wild type mice (Figure 5B). In contrast, *GALC*^*G*41*S*/G41*S*^ mice showed similar motor activities as per line crossing to wild type mice (Figure 5C). Interestingly, *GALC*^*G*41*S*/G41*S*^ mice showed higher time spent in the center quadrant at each time point of the study (Figure 5D).

### Spatial Learning Deficiencies Introduced by the G41S Mutation

**FIGURE 6:**
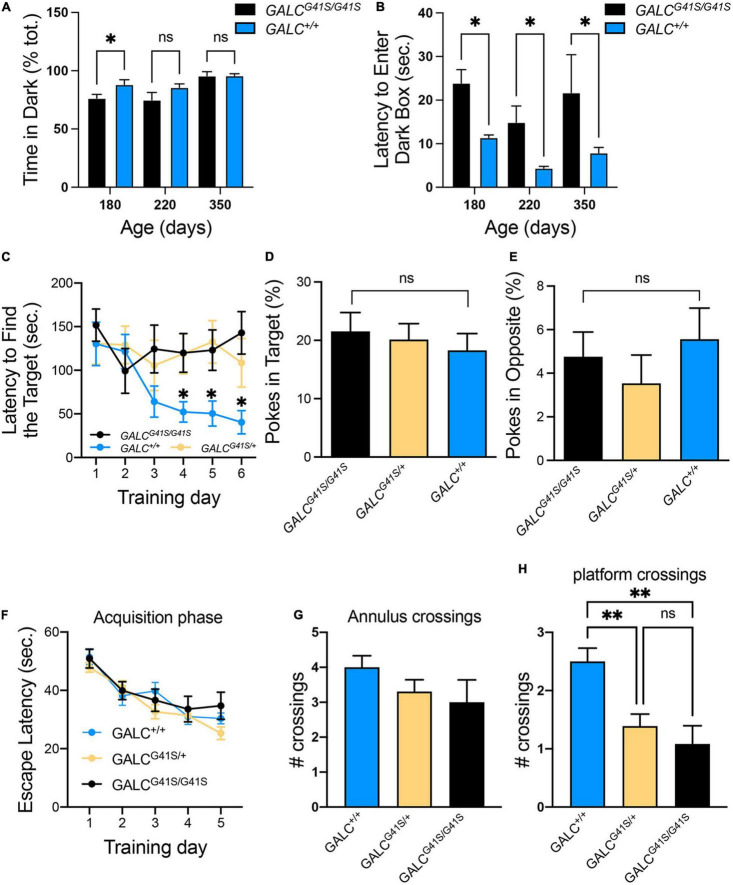
Cognitive capacity and spatial learning performance are slightly impaired in *GALC^G41S/G41S^* mice. **(A,B)** Activity of *GALC^G41S/G41S^* mice in the dark-light box showed slightly less time spent exploring the dark compartment in younger (P180) vs aged older (P220, P350), with an increased latency to enter in the dark box at all time points. Data represent the mean + SEM of *n* = 4–6/group, analyzed by ANOVA/Tukey’s *post hoc* test *p < 0.05. **(C–E)** The Barnes maze test showed *GALC^G41S/G41S^* and *GALC^G41S/+^* mice increased times to identify the target hole **(C)**, with no significant differences in the percentage of pokes in target **(D)** vs opposite **(E)** holes. Data represent the mean + SEM of *n* = 6/group, analyzed by ANOVA/Tukey’s *post hoc* test *p < 0.05. **(F–H)** The Morris water maze test measured no significant differences in the escape latency **(F)** and annulus crossings **(G)** but significant decreases in the number of platform crossings in both *GALC^G41S/G41S^* and *GALC^G41S/+^* mice. Data represent the mean + SEM of *n* = 6–8/group, analyzed by ANOVA/Tukey’s *post hoc* test ^**^p < 0.01.

The open field test showed increased exploratory behaviors of *GALC*^*G*41*S*/G41*S*^ mice. We wanted to investigate this in more detail by testing their response to the dark-light box. *GALC*^*G*41*S*/G41*S*^ mice were tested at 180, 220, and 350 days of age showing similar average time spent exploring the dark compartment, although younger P180 *GALC*^*G*41*S*/G41*S*^ mice spent less time in the darkness (Figure 6A). Remarkably, when the latency to enter the dark box was measured, *GALC*^*G*41*S*/G41*S*^ mice showed increased times spent in the lighted box at every time point (Figure 6B).

To assess whether spatial learning and memory functions are impacted in *GALC*^*G*41*S/G*41*S*^ mice, mice were tested in the Barnes and Water Morris maze tests. Interestingly, both homozygous and heterozygous mice for the *GALC*^*G*41*S/G*41*S*^ mutation showed higher latencies to find their target in the Barnes test, in contrast to wild type littermates which rapidly learned where the target was (Figure 6C). This might be supported by the lack of significant differences in poking the target (Figure 6D) or opposite (Figure 6E) holes. While there were no significant differences in the percentage of pokes in target and opposite holes, all genotypes spent more time exploring the target hole (∼20%) than the opposite hole (∼5%) (Figures 6D,E). When tested in the Morris water maze, all genotypes showed similar learning capacity to find the platform (Figure 6F). Likewise, no significant differences were observed in the number of annulus crossings between *GALC*^*G*41*S/G*41*S*^ and wild-type mice (Figure 6G). However, both homozygous *GALC*^*G*41*S/G*41*S*^ and heterozygous *GALC*^*G*41*S*/+^ mice showed a significant decrease in the number of crossings over the same exact location on the platform compared with wild-type controls (Figure 6H).

### Neuropathology in the Brains of *GALC*^*T*513*M*/T513*M*^ and *GALC*^*G*41*S*/G41*S*^ Mice

**FIGURE 7:**
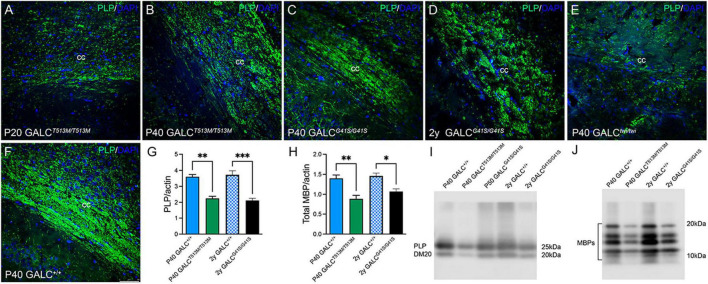
*GALC*^*T*513*M/T*513*M*^ and GALC^*G41S/G41S*^ mice exhibit signs of early and late central demyelination. Confocal microscopy after immunohistochemistry using anti-PLP detected reductions of PLP (in green) expression in the corpus callosum of *GALC^T513M/T513M^* mice at P20 **(A)** and P40 **(B)**, which are comparable to demyelination observed in P40 TWI **(E)**. In contrast, demyelination (indicated as a reduction of PLP immunofluorescence) was less evident at P40 **(C)**, and patchy in the 2 year old **(D)** *GALC^G41S/G41S^* corpus callosum. **(F)** shows PLP staining in P40 *GALC*^+/+^ corpus callosum. Blue: DAPI. Bar = 50 mm. **(G–J)** Semiquantitation of myelin PLP and MBP levels (by western blotting) in whole brain extracts. Data represent the mean + SEM of *n* = 4–6/group, analyzed by ANOVA/Tukey’s *post hoc* test *p < 0.05, ^**^p < 0.01, ^***^p < 0.005.

To examine central myelination, brain cryosections were stained for myelin proteolipids (PLP). We focused on the corpus callosum, a structure that is heavily demyelinated in the TWI brain at P40 (Figure 7E, compare with control levels in Figure 7F). *GALC*^*T*513*M*/T513*M*^ and *GALC*^*G*41*S/G*41*S*^ mice showed distinct patterns of central demyelination. *GALC*^*T*513*M/T*513*M*^ mice displayed the expected rapid and severe demyelination for a short lived leukodystrophic condition at P20 (Figure 7A) and P40 (Figure 7B) brains. Interestingly, demyelination appeared patchy in contrast to more global and diffuse damage observed in TWI brains. In contrast, while the level of PLP staining seemed undisturbed in P40 *GALC*^*G*41*S/G*41*S*^ mice (Figure 7C), it clearly showed signs of patchy demyelination by 2 years (Figure 7D). Examination at intermediate time points (P180 and P350) showed PLP staining comparable to WT (data not shown), suggesting that central demyelination is a late event in *GALC*^*G*41*S/G*41*S*^ mice. Loss of myelin was confirmed by measuring PLP (Figures 7G,I) and MBP (Figures 7H,J) levels on semiquatitative western blotting from P40 *GALC*^*T*513*M/T*513*M*^ and 2y *GALC*^*G*41*S/G*41*S*^ total brain extracts.

**FIGURE 8:**
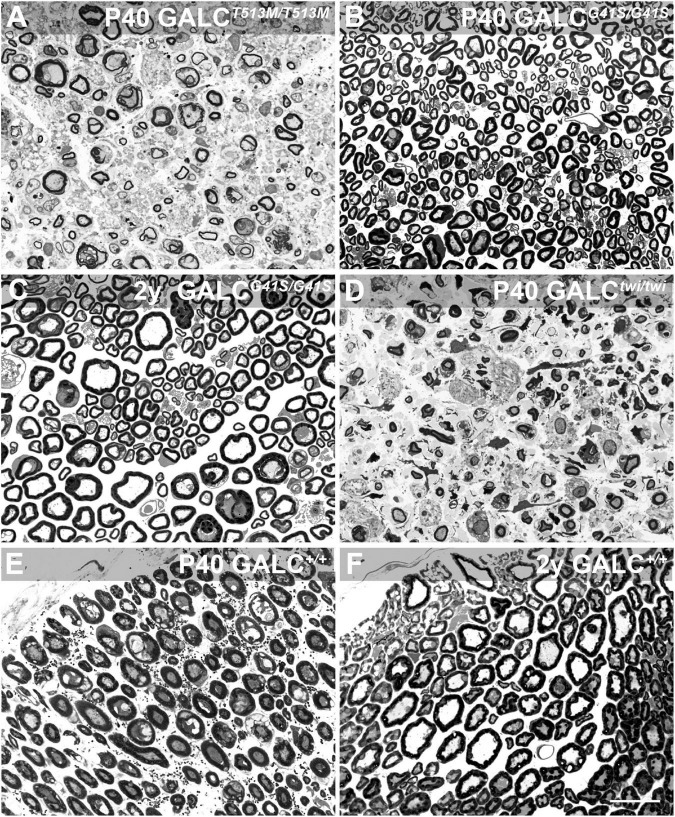
*GALC*^*T*513*M/T*513*M*^ but not GALC^*G41S/G41S*^ mice exhibit signs of peripheral demyelination. Toluidine blue staining on plastic cross sections of sciatic nerves showed pronounced demyelination and edema in nerves from P40 *GALC^T513M/T513M^* mice **(A)**, comparable to damage observed in nerves from age matched TWI mice **(D)**. In contrast, no significant damage to myelin was detected in nerves from either P40 **(B)** or 2 y-old **(C)** *GALC^G41S/G41S^* mice. **(E,F)** cross sections of P40 and 2y WT sciatic nerves. Bar = 20 μm.

Peripheral myelination also showed contrasting patterns of damage in *GALC*^*T*513*M/T*513*M*^ and *GALC*^*G*41*S/G*41*S*^ mice. Toluidine blue staining of sciatic nerve cross sections indicated high levels of peripheral demyelination (i.e., reduced thickness or complete loss of myelin sheaths in axonal profiles), edema (i.e., loss of packed myelinated axons, with swelling of extracellular spaces), and inflammation (i.e., presence of macrophagic profiles within the nerve) in the nerves of P40 *GALC*^*T*513*M/T*513*M*^ (Figure 8A) comparable to age-matched P40 TWI nerves (Figure 8D). In contrast, early (P40, Figure 8B) or aged (2y, Figure 8C) nerves from *GALC*^*G*41*S/G*41*S*^ mice did not show significant signs of demyelination or damage. Figures 8E,F show P40 and 2y WT nerves. These results suggest that both new mutants undergo distinct patterns of demyelination: while *GALC*^*T*513*M/T*513*M*^ mice suffer from rapid central and peripheral demyelination, *GALC*^*G*41*S/G*41*S*^ mice undergo mostly, if not solely, a late-onset central demyelination, without major impact on peripheral nerves.

**FIGURE 9:**
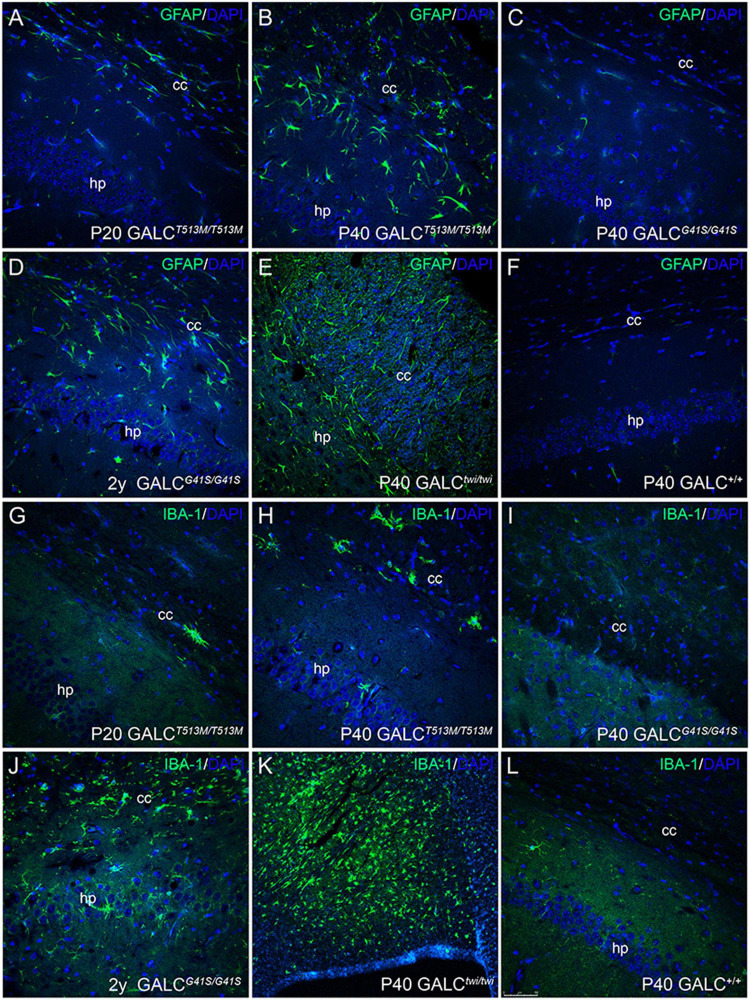
Inflammatory responses in the brain of *GALC*^*T*513*M/T*513*M*^ and GALC^*G41S/G41S*^ mice. Immunohistochemistry using anti-GFAP **(A–F)** and anti-IBA-1 **(G–L)** antibodies detected gliotic responses (in green) in the corpus callosum and hippocampus of *GALC^T513M/T513M^* and *GALC^G41S/G41S^* mice. Confocal microscopy showed that GFAP+ astroglial cells were comparable to those observed in TWI mice **(E)**. In contrast, IBA-1+ cells were less abundant in either mutant than in TWI mice **(K)**. Bar = 50 μm, except in panel **(K)**, where is 100 μm.

Inflammation was evaluated by immunostaining for GFAP and IBA-1, which identify activated astrocytes (Brenner, 2014) and microglia (Norden et al., 2016). As expected, GFAP+ astrogliosis was markedly increased in the brain of P40 *GALC*^*T*513*M/T*513*M*^ mice (Figure 9B), to levels comparable to those observed in P40 TWI (Figure 9E). Astrogliosis was also detectable in younger P20 *GALC*^*T*513*M/T*513*M*^ mice (Figure 9A), suggesting that inflammatory responses start early in the nervous system of *GALC*^*T*513*M/T*513*M*^ mice. Astrocyte responses in *GALC*^*G*41*S*^ mice were weaker than in *GALC*^*T*513*M/T*513*M*^ mice, with detectable GFAP+ astroglia in 2y *GALC*^*G*41*S/G*41*S*^ mice (Figure 9D). At earlier ages (P40), GFAP staining in *GALC*^*G*41*S/G*41*S*^ and WT brains was indistinguishable (Figures 9C,F). Interestingly, IBA-1 immunostaining was less intense in both mutants (Figures 9G–J) compared with the high levels detected in the P40 TWI brain (Figure 9K). Figure 9L shows IBA-1 in WT.

### Distinct Autophagolysosomal Responses in *GALC*^*T*513*M*/T513*M*^ and *GALC*^*G*41*S*/G41*S*^

**FIGURE 10:**
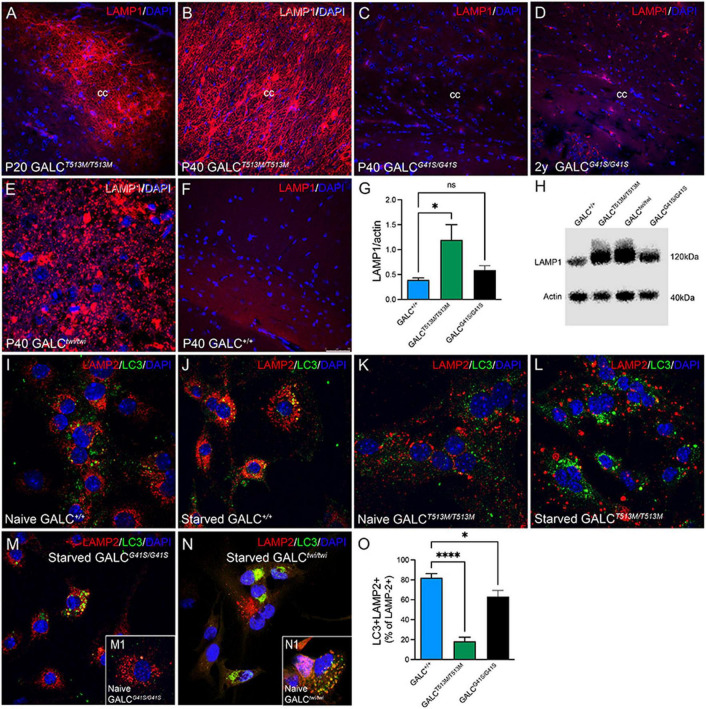
Increased Expression of LAMP-1 in White Matter Areas of *GALC*^*T*513*M/T*513*M*^ and GALC^*G41S/G41S*^ Mice and Decreased Autophagolysosome Fusion in Cultured Neural Precursors Immunohistochemistry using anti-LAMP-1 antibodies (in red) was used to evaluated lysosomal responses in white matter areas. Confocal microscopy detected very robust increases of LAMP-1 expression in interfascicular oligodendrocytes in white matter areas like the corpus callosum in P20 **(A)** and P40 **(B)** *GALC^T513M/T513M^* mice, to levels observed in TWI mice **(E)**. In contrast, LAMP-1 expression in *GALC^G41S/G41S^* oligodendrocytes was increased in aged mutants **(D)**, but not in younger P40 **(C)** mice. **(F)** shows LAMP-1 staining in P40 *GALC*^+/+^ corpus callosum. Blue stain: DAPI. Bar = 50 μm. **(G,H)** Semiquantitation of LAMP-1 levels (by western blotting) in whole brain extracts. Data represent the mean + SEM of *n* = 4/group, analyzed by ANOVA/Tukey’s *post hoc* test *p < 0.01; ns, not significant. **(I–L)** WT **(I,J)** and *GALC^T513M/T513M^* **(K,L)** cells were fed with our without (starved) 1% FBS for 4 h before double staining for LAMP-2 (red) and LC3 (green). **(M,N)** *GALC^G41S/G41S^* **(M)** and TWI **(N)** cells were also starved for 4 h before immunocytochemistry. Insets show naïve cells, respectively. **(O)** Confocal images were used for counting the total number of LAMP-2+ puncta and the number of double labeled LC3+/LAMP-2+ puncta. Data represent the mean + SEM of 15–20 cells/group, analyzed by ANOVA/Tukey’s *post hoc* test *p < 0.05, ^****^p < 0.001.

Alterations in lysosomal biogenesis are characteristic findings in KD (Marshall et al., 2018). This can be detected by increased expression of the lysosomal marker LAMP-1, as shown in the P40 TWI brain (Figure 10E). Immunostaining of LAMP-1 showed very intense signals in all white matter areas such as the corpus callosum in both P20 (Figure 10A) and P40 (Figure 10B) *GALC*^*T*513*M/T*513*M*^ mice. In contrast, white matter areas in the brain of *GALC*^*G*41*S/G*41*S*^ mice have weaker immunoreactions for LAMP-1, with levels (Figure 10C) comparable with WT (Figure 10F) at P40 and moderately increased in 2y *GALC*^*G*41*S/G*41*S*^ mice (Figure 10D). Increase of LAMP-1 was confirmed on semiquatitative western blotting from P40 *GALC*^*T*513*M/T*513*M*^ and 2y *GALC*^*G*41*S/G*41*S*^ total brain extracts (Figures 10G,H).

We and others have indicated deficiencies in the autophagic function in TWI mice (Ribbens et al., 2014; Del Grosso et al., 2019, 2022; Lin et al., 2020). To evaluate whether autophagy is also defective in the new *GALC*^*T*513*M/T*513*M*^ and *GALC*^*G*41*S/G*41*S*^ mice, subventricular zone-derived neural progenitor cultures (Felling et al., 2006; Givogri et al., 2008) were starved for 4 h (Mejlvang et al., 2018) before double immunocytochemistry for LC3 and LAMP-2. In normal conditions, starvation promotes autophagy which can be detected by increased fusion of autophagosomes with lysosomes as seen with WT cells (Figures 10I,J). As expected, TWI cells have increased production of lysosomes and autophagosomes, which remain largely segregated even when cells underwent starvation (compare Figure 10N with naïve cells in inset N1). Likewise, the basal level of LAMP-2+ lysosomes and LC3+ autophagosomes in naïve (non-starved) *GALC*^*T*513*M/T*513*M*^ cells is clearly increased (Figure 10K). Starvation only worsens this situation without significant fusion of both organelles (Figure 10L). Instead, formation of autophagolysosomes after starvation seems less compromised in *GALC*^*G*41*S/G*41*S*^ cells (Figure 10M, compare with naïve *GALC*^*G*41*S/G*41*S*^ cells, inset M1). Quantification of double labeled LC3+/LAMP-2+ puncta (as a percentage from the total number of LAMP-2+ puncta) confirmed these observations in starved cell cultures (Figure 10O).

**FIGURE 11:**
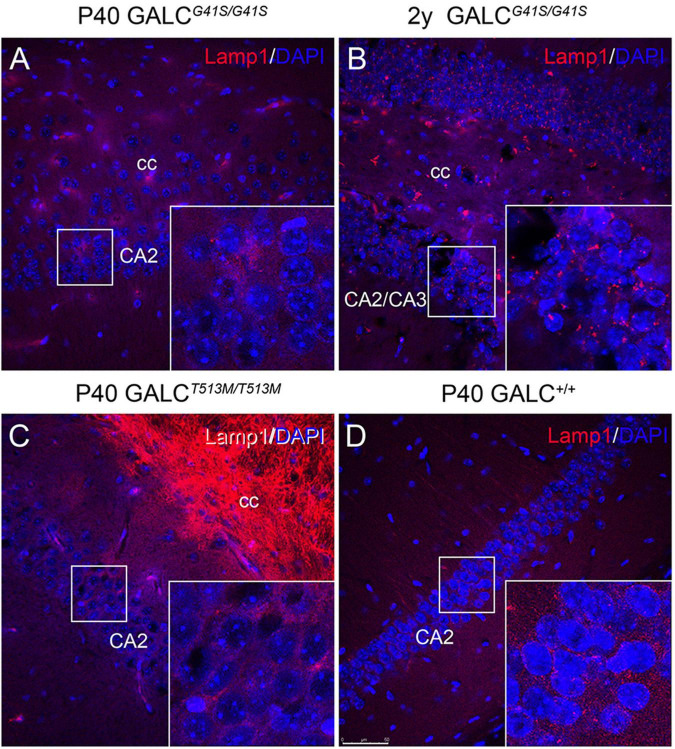
Increased expression of LAMP-1 in aged *GALC^G41S/G41S^* neurons. Confocal microscopy after immunohistochemistry using anti-LAMP-1 antibodies (in red) was used to evaluated lysosomal responses in hippocampal neurons. Lamp1 expression was not significantly affected in either P40 *GALC^G41S/G41S^* **(A)** or P40 *GALC^T513M/T513M^* **(C)** neurons. However, larger Lamp1+ puncta were detected in 2 year-old *GALC^G41S/G41S^* neurons **(B)**, in comparison to control levels **(D)**. Blue: DAPI. Bar = 50 μm.

LAMP-1 immunostaining in neuronal rich areas such as the hippocampus was markedly low in the *GALC*^*T*513*M/T*513*M*^ brain (Figure 11C), with patterns and levels comparable with WT (Figure 11D). In contrast, there was an apparent increase in LAMP-1+ puncta in aged *GALC*^*G*41*S/G*41*S*^ neurons (Figure 11B box) compared to levels observed at younger ages (Figure 11A box).

## Discussion

This study presents two new murine KD models carrying point mutations T513M, frequent in human patients with infantile forms of KD (Wenger et al., 1997), and G41S, found in many adult forms of KD (Lissens et al., 2007). The new mutants were generated using CRISPR-Cas9 gene editing and show remarkably reproducible phenotypes, with viable carrier breeders.

*GALC*^*T*513*M/T*513*M*^ mice show clinical signs of an early neurological condition, with onset of motor and behavioral declines at ∼P24. Mutant mice rapidly progress into stereotypic manifestations of a severe leukodystrophy, with body weight loss, twitching, tremor, and eventually death by the 7th week of life. This mutation is present in many infant patients of European ancestry, contributing to ∼10% of infant forms of KD (Kleijer et al., 1997). These patients show early-onset motor declines and premature death. Homozygous mice for *GALC*^*T*513*M/T*513*M*^ displayed the expected progression, and thus, constitute a newly engineered but genuine model for infantile KD, matching a severe mutation found in human patients. While the TWI mouse has been historically used as a model for infantile KD, it is more of a surrogate model for the 30 kb deletion found in infantile patients (Kleijer et al., 1997), because the deficiency of GALC activity in TWI mice is due to the absence of detectable GALC protein. In contrast, the new *GALC*^*T*513*M/T*513*M*^ mouse presents with a clinical disease that recapitulates that of the TWI but with detectable protein both by western blotting and immunohistochemistry.

Mice with the *GALC*^*G*41*S*^ mutation showed normal postnatal development, with no apparent compromise in survival, minimal changes in motor skills, and some alterations in anxiety-type behaviors measurable in aged (>P250) mutants. Patients with this mutation usually present with adult onset manifestations, with less neurological compromise, at least during the first several years of life (Lissens et al., 2007). Thus, the *GALC*^*G*41*S/G*41*S*^ mutant represents a genuine genetic model recapitulating many aspects of adult-onset KD.

The effects of each mutation on enzymatic activity and lysosomal translocation were remarkably different. *In vitro* studies have reported how different point mutations in the GALC gene can affect not only the activity, but also its trafficking to the lysosome (Shin et al., 2016; Irahara-Miyana et al., 2018). The *GALC*^*T*513*M*^ mutation changes a polar side chain to a hydrophobic side chain with loss of stabilizing hydrogen bond and steric hindrance (Deane et al., 2011). *In vitro* studies have shown that this change not only effectively abolishes GALC activity but also largely blocks the mutated protein from reaching the lysosomal compartment (Shin et al., 2016). Aligned with this, enzyme activity and localization analyses in our study show that the *GALC*^*T*513*M/T*513*M*^ mutated protein lacks detectable enzyme activity, and a large fraction of the protein remains outside of the lysosomal compartment.

The *GALC*^*G*41*S*^ mutation introduces a polar side chain in the TIM barrel domain of the 50 kDa GALC subunit (Lissens et al., 2007; Deane et al., 2011; Spratley et al., 2016; Hill et al., 2018), and thus should have minimal effect on the catalytic site or to induce major protein misfolding impairing transport to lysosomes (Shin et al., 2016). In fact, our *in vivo* studies show that >80% of GALC^*G*41*S*^ immunodetectable protein co-localized with Lamp2+ puncta, retaining significant residual enzyme activity. Likely, the translocation of GALC^*G*41*S*^ enzyme residual activity to lysosomes explains the lower accumulation of psychosine in this mutant. In contrast, the *GALC*^*T*513*M/T*513*M*^ mutants, which have poor lysosomal translocation of a mutated protein without residual enzyme activity, accumulated high levels of psychosine, comparable with levels measured in TWI mice.

The new KD mutants show interesting and chronologically different neuropathological changes. A direct correlation between toxic accumulations of psychosine and levels of demyelination, astrogliosis, and microglial dysfunction in the nervous system of the TWI mouse is well established (Ohno et al., 1993; Taniike and Suzuki, 1994; Giri et al., 2008; Kondo et al., 2011; Claycomb et al., 2014; O’Sullivan and Dev, 2015). As expected, these three neuropathological responses started at onset of clinical signs in *GALC*^*T*513*M/T*513*M*^ mutants and worsened as disease progressed. Demyelination, while impacting all levels of white matter in the nervous system of *GALC*^*T*513*M/T*513*M*^ mutants, appeared patchy and less diffuse than that observed in TWI mice, which may indicate undergoing segmental demyelination. Whether there is remyelination ongoing to repair demyelination is currently one area of study in our laboratory. In contrast, central demyelination and gliosis in *GALC*^*G*41*S*/G41*S*^ mutants were evident only in older mutants, consistent with late-onset neuropathology of adult forms of KD. Peripherally, both mutants also show different patterns of demyelination, which was clearly detected in sciatic nerves from *GALC*^*T*513*M/T*513*M*^ mutants but absent in *GALC*^*G*41*S*/G41*S*^ nerves. The different involvement of central and peripheral nerve damage in these mutants clearly and quantifiably place the two models into the early-onset (*GALC*^*T*513*M/T*513*M*^) and late-onset (*GALC*^*G*41*S/G*41*S*^) categories.

Behavioral examination of both mutants showed clear indications of early (*GALC*^*T*513*M/T*513*M*^) vs late (*GALC*^*G*41*S*/G41*S*^) motor involvement. *GALC*^*T*513*M/T*513*M*^ mutants showed stereotypic motor declines comparable to those measured in TWI mice (Olmstead, 1987). All motor readouts, including grip strength, ledge walking, rotarod, and open field tests indicated a progressive neurological involvement of motor pathways, leading to severe and rapid paralysis. On the other hand, *GALC*^*G*41*S*/G41*S*^ mutants showed mild and late onset deficits in some of the motor tests measured in our study. Interestingly, *GALC*^*G*41*S/G*41*S*^ mutants showed alterations in behavioral aspects involving exploratory activities and anxiety-related responses. Particularly, *GALC*^*G*41*S/G*41*S*^ mutants showed increased times involving exploratory activities in the open field, light/dark box, and the Barnes maze tests. This may suggest that *GALC*^*G*41*S/G*41*S*^ mutants are less anxious, and/or more curious. There are very few studies that have studied cognitive aspects on in KD, with most research focused on infantile cases and fewer on adult-onset cases (Patrick and Wilson, 1972; Crome et al., 1973; Loonen et al., 1985; Olmstead, 1987; Kolodny et al., 1991; Jardim et al., 1999; Debs et al., 2013; Xia et al., 2020; Yoon et al., 2021). In general, most neurodevelopmental deficits in KD have been associated with the loss of myelin and motor skills, which undoubtedly impacts on the capacity of the patient to reach neurodevelopmental milestones. The *GALC*^*G*41*S*^ mutation, which appears to trigger mild and later onset demyelination (Saavedra-Matiz et al., 2016), with minimal involvement of motor and learning skills, shows unexpected anxiolytic effects, at least in some exploratory tasks. Interestingly, changes in social behavior have been described occasionally in KD carriers (Christomanou et al., 1981). Considering that KD is a recessive trait, manifested only in homozygosis, the presence of subtle behavior changes in carriers of the *GALC*^*G*41*S*^ mutation may suggest some level of phenotypic variation in adult-onset KD, at least with some mutations. Defects in remyelination in heterozygous carriers of the twitcher mutation have been described (Scott-Hewitt et al., 2017, 2020), but nothing is known about the effects of GALC haploinsufficiency on cognition. Understanding the cause for these behavioral alterations is an exciting field of study which is under further examination in our laboratory, as it may be relevant to understand phenotypic manifestations reported in carriers for this and other rare conditions (Christomanou et al., 1980, 1981; Cheung and Ewens, 2006).

Impairment in lysosomal activity has been previously reported in several LSDs, with responses involving the proliferation of lysosomes (Napolitano et al., 2015; Sambri et al., 2017). Indeed, the TWI nervous system is characterized by an increased accumulation of lysosomes, detected by their high expression of LAMP-2 (Marshall et al., 2018) and by defective autophagy (Ribbens et al., 2014; Del Grosso et al., 2019, 2022). Both new mutants showed increased expression of lysosomal markers in the brain, although with marked differences. Expression of LAMP-1 is robustly upregulated in white matter in the brain of the *GALC*^*T*513*M*/T513*M*^ mutant since the onset of disease. In contrast, lysosomal responses in the *GALC*^*G*41*S/G*41*S*^ mutant in white matter areas are low and only detected in aged mutants. Aligned with results measured in TWI cells (Ribbens et al., 2014; Del Grosso et al., 2019, 2022), *GALC*^*T*513*M/T*513*M*^ and *GALC*^*G*41*S/G*41*S*^ cells also showed severe (*GALC*^*T*513*M/T*513*M*^) and mild (*GALC*^*G*41*S/G*41*S*^) defects in autophagic responses to starvation. Interestingly, aged *GALC*^*G*41*S*/G41*S*^ mutant neurons showed larger LAMP-1+ puncta, indicative of a late-onset lysosomal dysfunction in this mutant. This late-onset lysosomal response may undermine cellular functions in *GALC*^*G*41*S/G*41*S*^ mutant neurons and lead to -some- of the observed alterations in exploratory behavior in these mice. Clinical manifestations affecting behavior and cognition have been described in several inborn errors of the metabolism (Gutschalk et al., 2004; Cox, 2020). Whether the *GALC*^*G*41*S*^ mutation, and perhaps others, affecting the GALC gene directly or indirectly impact the structure and/or activity of synapses of neural networks involved in higher cognitive functions remains an open question (Castelvetri et al., 2011; Cantuti Castelvetri et al., 2013; Cantuti-Castelvetri and Bongarzone, 2016; Marshall and Bongarzone, 2016; Gowrishankar et al., 2020; Rebiai et al., 2021) and is the subject of a follow up study in our laboratories. In addition to changes in the metabolism of psychosine, other factors such as accumulation of additional metabolites like lactosylceramide (Papini et al., 2022), alteration of other signaling pathways following lipid raft rearrangements in membranes (White et al., 2009; Sural-Fehr et al., 2019; Belleri and Presta, 2022), abnormal microglia-mediated synaptic pruning (Sellgren et al., 2019), could be also contributors to changes in behavioral responses.

The new *GALC*^*T*513*M/T*513*M*^ and GALC^*G*41*S/G*41*S*^ murine models described in this study offer contrasting yet unique opportunities to study pathogenic mechanisms affecting synaptic and myelin function and their contribution to the spectrum of phenotypes observed in KD. These new transgenic lines appear as bona fide models of infantile and adult-onset KD, with evident advantages for their use in pre-clinical therapeutic studies. Importantly, this study shows the potential of using CRISPR-Cas9 gene editing to introduce and study functional effects of other pathogenic mutations in cis or trans with specific single nucleotide polymorphisms (SNPs) described in the *GALC* gene (Saavedra-Matiz et al., 2016). For example some SNPs such as p.I546T, while not pathogenic *per se*, are known to modify the overall GALC activity when segregated with point mutations such as T513M. This gene editing approach will likely be crucial to help understand genotype-phenotype correlations and mechanisms defining an early vs a late manifestation of Krabbe disease.

## Data Availability Statement

The original contributions presented in the study are included in the article/supplementary material, further inquiries can be directed to the corresponding author.

## Ethics Statement

The animal study was reviewed and approved by Animal Care and Use Committee at the University of Illinois at Chicago.

## Author Contributions

RR performed histology, immunocytochemistry, and wrote manuscript. ER and RB performed quantitative LC-MS-MS of psychosine. SZ and LT performed Morris water maze and wrote dedicated sections. DN established founders. GS and MJ performed cell culture experiments. RF performed motor behaviors. BW and SB performed monoclonal production and wrote dedicated sections. XJ and MS performed isobaric LC-MS-MS and wrote dedicated sections. MG performed histology and wrote dedicated sections. EB designed the study, performed confocal microscopy, and wrote, edited, and proofedited the manuscript. All authors contributed to the article and approved the submitted version.

## Conflict of Interest

The authors declare that the research was conducted in the absence of any commercial or financial relationships that could be construed as a potential conflict of interest.

## Publisher’s Note

All claims expressed in this article are solely those of the authors and do not necessarily represent those of their affiliated organizations, or those of the publisher, the editors and the reviewers. Any product that may be evaluated in this article, or claim that may be made by its manufacturer, is not guaranteed or endorsed by the publisher.
